# Differences in infant feeding methods at one month postpartum among women with psychiatric disorders and factors influencing exclusive breastfeeding

**DOI:** 10.1186/s13034-025-00973-7

**Published:** 2025-10-27

**Authors:** Ayumi Kuramitsu, Kazutaka Ohi, Shunsuke Sugiyama, Tomomi Shiga, Tatsuro Furui, Toshiki Shioiri

**Affiliations:** 1 Department of Psychiatry, Gifu University Graduate School of Medicine, 1-1 Yanagido, Gifu, 501-1194 Japan; 2 Department of Obstetrics and Gynecology, Gifu University Graduate School of Medicine, Gifu, Japan; 3 Center for Perinatal and Reproductive Medicine, Gifu University Graduate School of Medicine, Gifu, Japan

**Keywords:** Infant feeding methods, Breastfeeding, Schizophrenia, Antipsychotic, Benzodiazepine, Polypharmacy, Postpartum

## Abstract

**Background:**

Women with psychiatric disorders tend to have lower rates of exclusive breastfeeding despite of the benefits. Maternal use of psychotropic drugs has been suggested to influence their infant feeding methods. In this study, we retrospectively investigated differences in infant feeding methods (exclusive breastfeeding, formula feeding, and mixed feeding) among mothers with psychiatric disorders one month postpartum (*n* = 149), as well as the factors influencing infant feeding methods.

**Methods:**

Data on the infant feeding methods of individuals with schizophrenia spectrum disorders (SSDs, *n* = 32), bipolar disorders (BDs, *n* = 23), depressive disorders (DDs, *n* = 33), and anxiety disorders, and stress-related disorders (ASRDs, *n* = 61) were extracted from medical records at a single institute between 2008 and 2024. Differences in infant feeding methods among the disorder groups, and the influences of individual factors, including the regular use of psychotropic drugs at the time of childbirth, as well as newborn information, on the infant feeding methods were assessed.

**Results:**

Among the diagnostic groups, significant differences in infant feeding methods were observed (*F* = 6.52, *p* = 3.64 × 10⁻⁴), with a higher rate of formula feeding in the SSD group (72%) than in the other groups (BDs, 35%; DDs, 24%; ASRDs, 35%). In the SSD group, formula feeding was significantly correlated with the presence of antipsychotic (*beta* = 0.52, *p* = 2.93 × 10^− 3^). In the non-SSD group, formula feeding was significantly correlated with the regular use of benzodiazepines (*beta* = 0.43, *p* = 3.46 × 10^− 6^), the number of benzodiazepines used(*beta* = 0.38, *p* = 3.71 × 10^− 5^), the diazepam equivalent (*beta* = 0.31, *p* = 6.78 × 10^− 4^), and the number of psychotropic drugs used (*beta* = 0.41, *p* = 8.42 × 10^− 6^).

**Conclusion:**

Individuals with SSDs were more likely to choose formula feeding because of the use of antipsychotics at the time of childbirth, whereas individuals with non-SSDs tended to choose formula feeding because of psychotropic polypharmacy, including the use of benzodiazepines. These findings suggest that providing education on the safety of antipsychotics for patients with SSDs and avoiding polypharmacy in non-SSD patients might help promote exclusive breastfeeding.

**Supplementary Information:**

The online version contains supplementary material available at 10.1186/s13034-025-00973-7.

## Introduction

The importance of exclusive breastfeeding is well-established due to its nutritional, immune support, and long-term health benefits, such as child socioemotional and neurocognitive development [1–6]. The World Health Organization (WHO), and the American Academy of Pediatrics (AAP) recommend exclusive breastfeeding for the first six months after birth [6, 7]. Globally, 37–48% of women exclusively breastfeed for the first six months [8–11]. Exclusive breastfeeding is associated with better maternal mental health outcomes, such as decreased risks of depression and anxiety symptoms [12, 13], via biological factors including cortisol [14–16], oxytocin [14, 15, 17], progesterone, and estrogen levels [15], and psychological factors, such as maternal self-efficacy [18].

Schizophrenia spectrum disorders (SSDs) including schizophrenia, and schizoaffective disorder, bipolar disorders (BDs), depressive disorders (DDs), and anxiety disorders, and stress-related disorders (ASRDs) including anxiety disorders, adjustment disorder and dissociative disorder are major psychiatric disorders treated in the psychiatric departments of general hospitals. The age of onset for these disorders is between 10 and 30 years [19], and they often occur before pregnancy. The general fertility rate for women with psychiatric disorders appears to be increasing modestly [20, 21]. The presence of a psychiatric disorder is a serious factor considered during the perinatal period. SSDs and BDs are associated with the development of postpartum psychosis [22–25], and the psychotic symptom relapse rate in the postpartum period is approximately 35% [25]. DDs and ASRDs during the perinatal period are common, with a prevalence of approximately 20% for DDs and 10% for ASRDs [26–28]. As the exacerbation or relapses of psychiatric disorders can negatively impact an individual during the perinatal period as well as their child’s development [29–31], continuous psychotropic medication is recommended during the perinatal period [32].

Exclusive breastfeeding is also recommended for individuals with psychiatric disorders rather than formula feeding, and each individual should be supported in the choice of the infant feeding method that best suits them and their family [6, 32]. However, in individuals with psychiatric disorders, including SSDs, BDs, DDs and ASRDs, low rates of exclusive breastfeeding and inadequate support have been reported [33–38]. Individuals with SSDs and BDs are particularly likely to take multiple psychotropic medications [39], and it has been reported that individuals with SSDs have a lower intention to breastfeed than those the general population [38]. Maternal use of psychotropic drugs, such as antipsychotics, antidepressants, and mood stabilizers, has been suggested to influence exclusive breastfeeding practices [33, 40]. Among individuals with psychiatric disorders, more than a quarter reported that they were advised to stop exclusive breastfeeding because of the psychotropic drugs they were taking [40]. In addition, some side effects of psychotropic drugs, such as drowsiness, in individuals with psychiatric disorders might interfere with exclusive breastfeeding [40, 41].

Although the use of psychotropic drugs varies by psychiatric disorder, there is no research comparing infant feeding methods among postpartum women with different psychiatric disorders. Identifying differences in infant feeding methods among individuals with psychiatric disorders and the factors that influence these choices is important for implementing effective breastfeeding support strategies for postpartum women with psychiatric disorders. We hypothesized that infant feeding methods would differ among postpartum women with psychiatric disorders, e.g., the ratio of formula feeding in individuals with SSDs would be the highest across individuals with other psychiatric disorders because a greater percentage of individuals with SSDs are taking multiple psychotropic medications. Furthermore, we hypothesized that higher dosages or a greater number of psychotropic medication types may influence the increase in formula feeding rates among individuals. In this study, we retrospectively investigated differences in infant feeding methods, namely, exclusive breastfeeding, formula feeding, and mixed feeding, among individuals with psychiatric disorders (*n* = 149), and the factors influencing the infant feeding methods by psychiatric disorders.

## Methods

### Study design and subjects

This was a retrospective observational study in which data were collected from existing medical records at Gifu University Hospital in Japan. A total of 149 pregnant women with psychiatric disorders who delivered at this hospital between April 1, 2008, and March 31, 2024, were included. The participants resided in the Chubu region in Japan, and they were Japanese, with the exception of four individuals: two Chinese individuals, one Filipino individual, and one Brazilian individual. All the subjects had a primary diagnosis based on the Diagnostic and Statistical Manual of Mental Disorders, Fifth Edition, Text Revision (DSM-5-TR) [19]: SSDs (*n* = 32), BDs (*n* = 23), DDs (*n* = 33), and ASRDs (*n* = 61). The SSD group included individuals with schizophrenia (*n* = 29), brief psychotic disorder (*n* = 2), and schizoaffective disorder (*n* = 1). The BD group included individuals with bipolar I disorder (*n* = 6), and bipolar II disorder (*n* = 17). The DD group included individuals with major depressive disorder (*n* = 29), and persistent depressive disorder (*n* = 4). The ASRD group included individuals with panic disorder (*n* = 31), generalized anxiety disorder (*n* = 9), specific phobia (*n* = 1), obsessive compulsive disorder (*n* = 3), adjustment disorder (*n* = 8), dissociative disorders (*n* = 7), conversion disorder (*n* = 1), and somatic symptom disorder (*n* = 1). No participants in this study had comorbid substance use disorders. Each subject was diagnosed by psychiatrists, and in this study, the last primary diagnosis during the lifetime was extracted. The infant feeding methods for postpartum infants and individuals and their newborn information were extracted from medical records. The demographic details are summarized in Table 1. This study was performed in accordance with the World Medical Association’s Declaration of Helsinki and was approved by the Research Ethics Committees of Gifu University (2024-050). The opportunity to refuse by opting out was guaranteed.

**Table 1:** Demographic information of the individuals with psychiatric disorders and their newborns

	Total participants		Schizophrenia spectrum disorders	Bipolar disorders	Depressive disorders	Anxiety and Stress related disorders		
	(*n* = 149)		(*n* = 32)	(*n* = 23)	(*n* = 33)	(*n* = 61)	*χ*^*2*^ or *F* value	*p* value
Individual information								
Comorbid psychiatric disorders (+/-)	19/130		3/29	3/20	4/29	9/52	0.6 ^a^	0.91
Comorbid physical disorders (+/-)	37/112		4/28	5/18	8/25	20/41	4.8 ^a^	0.19
Physical complications related to pregnancy (+/-)	63/86		14/18	7/16	17/16	25/36	2.5 ^a^	0.47
Delivery methods (Vaginal delivery/Elective CS/Emergency CS)	110/24/15		18/8/6	16/5/2	28/3/2	48/8/5	8.7 ^a^	0.19
Age at childbirth (years)	32.2 ± 5.4		33.5 ± 5.7	32.5 ± 4.6	32.9 ± 5.2	30.8 ± 5.4	2.4	0.07
Pregnancy (first/2 or more)	95/54		19/13	14/9	21/12	41/20	0.7 ^a^	0.88
Occupation at the time of childbirth (+/-)	29/120		3/29	5/18	10/23	11/50	4.7 ^a^	0.20
Unmarried at the time of childbirth (+/-)	10/139		4/28	2/21	1/32	3/58	2.9 ^a^	0.41
Current smoker at the time of childbirth (+/-)	10/139		8/24	0/23	0/33	2/59	22.3 ^a^	**5.72 × 10** ^**− 5**^
Psychotropic drugs information								
Regular use of antipsychotics at the time of childbirth (+/-)	42/107		28/4	7/16	5/28	2/59	76.5 ^a^	**1.77 × 10** ^**− 16**^
Regular use of antidepressants at the time of childbirth (+/-)	27/112		2/30	1/22	7/16	17/44	10.1^a^	**0.018**
Regular use of benzodiazepine at the time of childbirth (+/-)	31/118		7/25	6/17	6/27	12/49	0.4 ^a^	0.94
Regular use of mood stabilizers at the time of childbirth (+/-)	11/138		2/30	7/16	1/32	1/60	21.8 ^a^	**7.21 × 10** ^**− 5**^
CPZeq at the time of childbirth (mg/day)	81.4 ± 172.4		280.5 ± 206.5	98.0 ± 222.3	23.0 ± 59.6	2.9 ± 15.8	32.5	**4.13 × 10** ^**− 16**^
IMIeq at the time of childbirth (mg/day)	18.0 ± 47.5		5.5 ± 21.4	3.3 ± 15.3	31.7 ± 72.4	22.8 ± 44.9	2.7	**0.049**
DZPeq at the time of childbirth (mg/day)	2.6 ± 7.5		2.4 ± 5.7	6.2 ± 14.2	2.3 ± 6.8	1.4 ± 3.2	2.3	0.084
Maximum RID among psychotropic drugs at the time of childbirth (%)	3.5 ± 3.9		6.1 ± 3.6	7.1 ± 4.1	2.0 ± 5.1	1.9 ± 3.8	9.1	**1.66 × 10** ^**− 5**^
Number of psychotropic drugs at the time of childbirth	1.0 ± 1.4		1.7 ± 1.5	1.3 ± 1.3	0.9 ± 0.4	0.6 ± 0.4	4.9	**2.86 × 10** ^**− 3**^
Number of antipsychotics at the time of childbirth	0.3 ± 0.6		1.0 ± 0.5	0.4 ± 0.7	0.2 ± 0.4	0.03 ± 0.2	39.3	**1.16 × 10** ^**− 18**^
Number of antidepressants at the time of childbirth	0.2 ± 0.5		0.09 ± 0.4	0.04 ± 0.2	0.3 ± 0.7	0.3 ± 0.5	2.7	**0.048**
Number of benzodiazepines at the time of childbirth	0.3 ± 0.7		0.2 ± 0.5	0.4 ± 0.7	0.3 ± 0.9	0.2 ± 0.5	0.4	0.77
Number of mood stabilizers at the time of childbirth	0.1 ± 0.2		0.09 ± 0.4	0.4 ± 0.5	0.03 ± 0.2	0.02 ± 0.1	8.3	**4.03 × 10** ^**− 5**^
Newborn information								
Gestational age at birth (weeks)	38.5 ± 5.4		38.4 ± 1.4	38.9 ± 1.4	38.3 ± 1.7	38.5 ± 1.5	0.7	0.55
Multiple gestation status (+/-)	2/147		2/30	0/23	0/33	0/61	7.4 ^a^	0.06
Sex of the newborn (male/female)	73/78		19/15	10/13	9/24	35/26	4.9 ^a^	0.18
Birth weight of the newborn (g)	2965.3 ± 466.3		3039.1 ± 571.3	2983.2 ± 438.9	2816.5 ± 372.9	2998.0 ± 434.3	2.1	0.11
Hospitalization in the NICU (+/-)	92/59		27/7	14/9	21/12	30/31	7.6 ^a^	0.06
At 1 min: Apgar score at 1 min	8.2 ± 0.9		8.1 ± 1.0	8.4 ± 0.9	8.3 ± 0.8	8.3 ± 0.9	1.9	0.13
At 5 min: Apgar score at 5 min	9.2 ± 0.8		9.1 ± 0.8	9.4 ± 0.6	9.2 ± 0.5	9.3 ± 0.9	2.6	0.06

*CS* Cesarean section,* CPZeq* chlorpromazine equivalent,* IMIeq* imipramine equivalent,* DZPeq* diazepam equivalent,* RID* relative infant dose,* NICU* neonatal intensive care unit. *P* < 0.05 is shown in bold. ^a^*χ*^*2*^ test

### Subject and newborn information related to pregnancy

The infant feeding methods, exclusive breastfeeding and formula feeding, were determined at each participant’s one-month postpartum visit. Furthermore, an intermediate feeding method that included both exclusive breastfeeding and formula feeding was defined as mixed feeding. Additionally, individual information on perinatal comorbid psychiatric disorders such as neurodevelopmental disorders, and personality disorders; comorbid physical disorders such as cardiovascular disorders, respiratory disorders, and endocrine disorders; physical complications related to pregnancy such as pregnancy-induced hypertension, gestational diabetes, and threatened preterm labor; delivery methods (vaginal delivery/elective cesarean section /emergency cesarean section); age at childbirth; first pregnancy status; occupational status at the time of childbirth; marital status at the time of childbirth; and smoking status during pregnancy was extracted from medical records. Furthermore, their newborn information, including the week of delivery, multiple gestation status, sex, birth weight, hospitalization in the neonatal intensive care unit (NICU), and Apgar score at 1 and 5 min was extracted from medical records.

### Psychotropic drug information

The data concerning the regular use of psychotropic drugs at the time of childbirth in individuals with psychiatric disorders were extracted from medical records. In this study, the regular use of psychotropic drugs at the time of childbirth was defined as the uninterrupted use of prescribed psychotropic drugs for at least four consecutive weeks immediately prior to delivery, as confirmed by the medical records. The presence and number of regular use of antipsychotics, antidepressants, benzodiazepines, and mood stabilizers at the time of childbirth were recorded. The antipsychotics included atypical antipsychotics, such as olanzapine, risperidone, quetiapine, aripiprazole, perospirone, blonanserin, and asenapine, and typical antipsychotics, such as chlorpromazine, haloperidol, and sulpiride. The antidepressants included selective serotonin reuptake inhibitors, such as sertraline, escitalopram, paroxetine, and fluvoxamine, serotonin-norepinephrine reuptake inhibitors, such as duloxetine, venlafaxine, milnacipran, noradrenergic and specific serotonergic antidepressants including mirtazapine, and trazodone. Benzodiazepines were considered, whereas nonbenzodiazepines, such as zolpidem, zopiclone, and eszopiclone, were not considered in this study. The mood stabilizers included lithium carbonate, carbamazepine, and lamotrigine. Valproic acid was excluded from the mood stabilizer category as it was not prescribed to any of the participants. We did not assess any as-needed medications for psychiatric conditions. Psychotropic dose equivalents were calculated using equivalency tables specifically developed for Japanese patients [42]. Chlorpromazine equivalents (CPZeq) were used for antipsychotics, imipramine equivalents (IMIeq) for antidepressants, diazepam equivalents (DZPeq) for benzodiazepines. These equivalents were used to standardize dosages across different drugs and were treated as continuous variables in the analysis. In each subject, the maximum relative infant dose (RID) value among the psychotropic drugs regularly used was extracted on the basis of the 2019 manual of lactational pharmacology on medications and mothers’ milk [43, 44].

### Statistical analysis

All the statistical analyses were performed using IBM SPSS Statistics 30.0 software (IBM Japan, Tokyo, Japan). To assess differences among the four disorder groups (SSD, BD, DD, and ASRD groups), categorical variables, such as the comorbidity of psychiatric disorders and the regular use of antipsychotics at the time of childbirth, were analyzed using chi-square tests, whereas continuous variables, such as the number of antipsychotics used at the time of childbirth and Apgar score at 5 min, were analyzed using analysis of variance (ANOVA). To investigate whether there were differences in infant feeding methods among the four disorder groups (SSD, BD, DD, and ASRD groups), an analysis of covariance (ANCOVA) was conducted with the infant feeding methods (exclusive breastfeeding, mixed feeding, and formula feeding) at one month postpartum as the dependent variable; diagnosis (SSD, BD, DD, and ASRD) as the independent variable; and age and year of childbirth as covariates. *Post-hoc* pairwise comparisons were conducted between disorder groups.

Next, to examine the association between the infant feeding methods at one month postpartum and an SSD diagnosis, a linear regression analysis was conducted with the infant feeding methods at one month postpartum as the dependent variable, the SSD and non-SSD diagnoses as the independent variables, and age and year of childbirth as covariates. Furthermore, to identify factors affecting the infant feeding methods at one month postpartum within the SSD group (*n* = 32) and non-SSD group (*n* = 117), a linear regression analysis was conducted with the infant feeding methods at one month postpartum (formula feeding: 3 > mixed feeding: 2 > exclusive breastfeeding: 1) as the dependent variable, individual and newborn variables as the independent variables, and age and year of childbirth as covariates.

The nominal two-tailed significance level for all the statistical tests was set at *p* < 0.05. To avoid type I error, the final significance level was set at *p* < 2.94 × 10^− 3^ (*α* = 0.05/17 independent variables because several variables, such as the Apgar scores at 1 and 5 min, and the regular use of antipsychotics at the time of childbirth and the number of antipsychotics used at the time of childbirth, were highly correlated) after applying Bonferroni correction.

## Results

### Differences in infant feeding methods at one month postpartum among the four disorder groups

We assessed differences in individual variables and newborn variables among the four disorder groups (SSD, BD, DD, and ASRD groups) (Table 1). Significant group differences were detected in smoking status at the time of childbirth (*χ*^*2*^ = 22.3, *p* = 5.72 × 10⁻^5^) and in the use of psychotropic drugs during childbirth (regular use of antipsychotics, *χ*^*2*^ = 76.5, *p* = 1.77 × 10⁻^16^; regular use of antidepressants, *χ*^*2*^ = 10.1, *p* = 0.018; regular use of mood stabilizers, *χ*^*2*^ = 21.8, *p* = 7.21 × 10⁻^5^; chlorpromazine equivalent, *F*_*3,145*_=32.5, *p* = 4.13 × 10⁻^16^; imipramine equivalent, *F*_*3,145*_=2.7, *p* = 0.049; maximum RID, *F*_*3,136*_=9.1, *p* = 1.66 × 10⁻^5^; number of psychotropic drugs, *F*_*3,145*_=4.9, *p* = 2.86 × 10⁻^3^; number of antipsychotics, *F*_*3,145*_=39.3, *p* = 1.16 × 10⁻^18^; number of antidepressants, *F*_*3,145*_=2.7, *p* = 0.048; number of mood stabilizers, *F*_*3,145*_=8.3, *p* = 4.03 × 10⁻^5^) (Table 1).

**Fig. 1:**
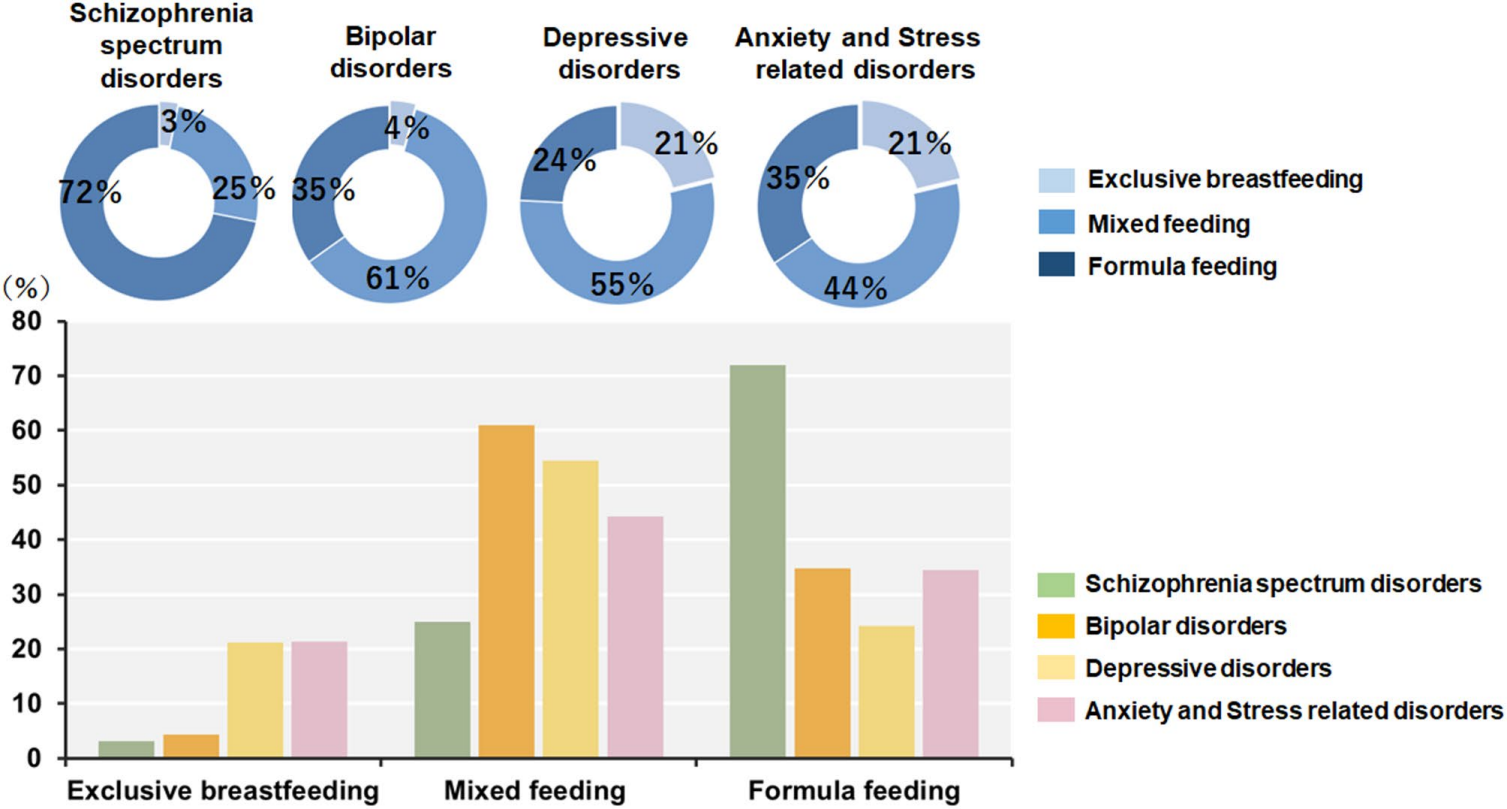
Infant feeding methods at one month postpartum in individuals with different psychiatric disorders. The Donat pie chart shows the percentage of infant feeding methods by individuals with psychiatric disorders, whereas the bar chart shows the percentage of psychiatric disorders by infant feeding methods

We investigated differences in infant feeding methods (exclusive breastfeeding, mixed feeding, and formula feeding) at one month postpartum among individuals with SSDs, BDs, DDs, and ASRDs (Fig. 1). Significant differences were observed among the disorder groups (*F*_*3,143*_=6.52, *p* = 3.64 × 10⁻⁴). In the *post-hoc* comparison of infant feeding methods among diagnostic groups (Supplementary Table 1), there were significant differences between the SSD group and the other groups: the BD group (*p* = 0.040), the DD group (*p* = 9.41 × 10^− 5^), and the ASRD group (*p* = 2.41 × 10^− 4^). The rate of formula feeding in the SSD group (72%) was greater than that in the other disorder groups (BD group, 35%; DD group, 24%; ASRD group, 35%), whereas the rates of mixed feeding in the other disorder groups (BD group, 61%; DD group, 55%; ASRD group, 44%) were greater than those in the SSD group (25%). An SSD diagnosis was significantly positively correlated with formula feeding (formula feeding > mixed feeding > exclusive breastfeeding) (*beta* = 0.33, *p* = 6.49 × 10^− 5^).

### Possible factors influencing infant feeding methods in the SSD group

**Fig. 2:**
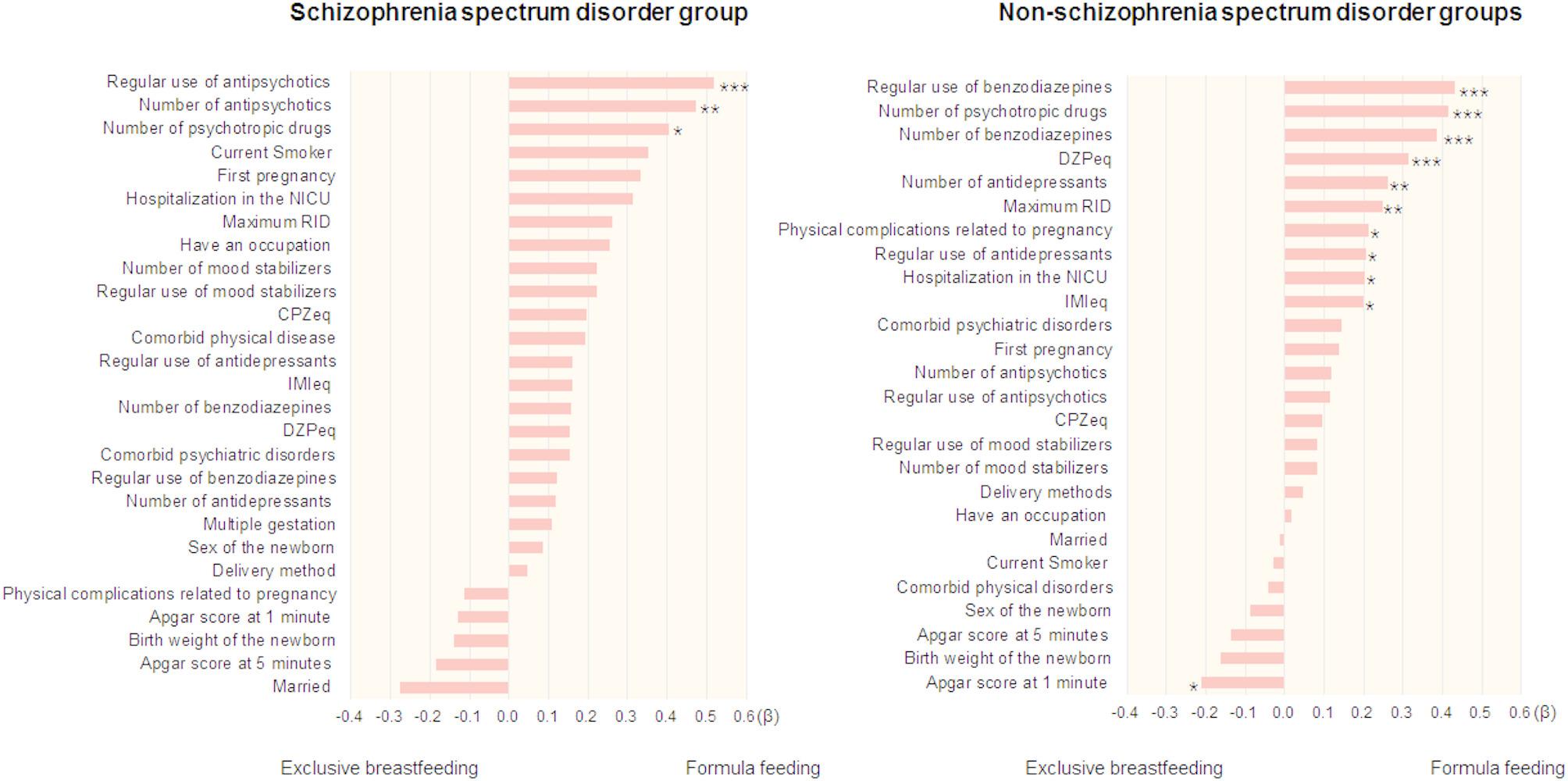
Factors influencing infant feeding methods in individuals with and without schizophrenia spectrum disorders. The *beta* of each factor was sorted according to the size of the *beta*. *** *p* < 2.94 × 10^− 3^, **p < 0.01, *p < 0.05. NICU, neonatal intensive care unit; RID, relative infant dose; CPZeq, chlorpromazine equivalent; IMIeq, imipramine equivalent; DZPeq, diazepam equivalent

Since the SSD group presented significant differences in infant feeding methods compared to those with the other disorder groups, we next focused on the SSD group, and examined possible clinical factors influencing infant feeding methods, including individual characteristics, newborn-related factors, and the use of psychotropic drugs, in the SSD group (Fig. 2, Supplementary Table 2). In the SSD group (*n* = 32), the formula feeding method at one month postpartum was significantly correlated with the use of antipsychotics (*beta* = 0.52, *p* = 2.93 × 10^− 3^), and was nominally correlated with the number of antipsychotics (*beta* = 0.47, *p* = 7.94 × 10^− 3^) and the number of psychotropic drugs (*beta* = 0.40, *p* = 0.028) used at the time of childbirth. Even after adjusting for psychiatric or physical comorbidities as covariates, our main findings remained unchanged (*p* < 0.05). However, no significant differences were found in any other psychotropic factors, such as the chlorpromazine equivalent, maximum RID of drugs used at the time of childbirth, or current smoking status (Fig. 2, Supplementary Table 2, *p* > 0.05).

### Possible factors influencing infant feeding methods in the non-SSD group

We additionally examined factors influencing infant feeding methods in the non-SSD group (Fig. 2, Supplementary Table 3). The formula feeding method at one month postpartum in the non-SSD group (*n* = 117) was significantly affected by the regular use of benzodiazepines (*beta* = 0.43, *p* = 3.46 × 10^− 6^), the number of psychotropic drugs used (*beta* = 0.41, *p* = 8.42 × 10^− 6^), the number of benzodiazepines used (*beta* = 0.38, *p* = 3.71 × 10^− 5^) and the DZPeq (*beta* = 0.31, *p* = 6.78 × 10^− 4^), and it was nominally affected by the number of antidepressants used (*beta* = 0.26, *p* = 5.50 × 10^− 3^), the maximum RID (*beta* = 0.25, *p* = 0.010), physical complications related to pregnancy (*beta* = 0.21, *p* = 0.026), the Apgar score at 1 min (*beta*=-0.21, *p* = 0.027), the use of antidepressants (*beta* = 0.21, *p* = 0.030), hospitalization in the NICU (*beta* = 0.20, *p* = 0.031) and the imipramine equivalent (*beta* = 0.20, *p* = 0.037). No significant correlations were found for the other factors (Fig. 2, Supplementary Table 3, *p* > 0.05).

## Discussion

To our knowledge, this study is the first to investigate the differences in infant feeding methods (exclusive breastfeeding, mixed feeding, and formula feeding) among individuals with psychiatric disorders (SSDs, BDs, DDs, and ASRDs) and their associations with clinical factors, including individual characteristics, newborn-related factors, and the use of psychotropic drugs. We found that infant feeding methods differed among individuals with psychiatric disorders, with higher rates of formula feeding and lower rates of exclusive breastfeeding and mixed feeding in individuals in the SSD group compared to the other psychiatric disorder groups. Therefore, participants were divided into those with SSDs and those with other psychiatric disorders. In the SSDs group, formula feeding was significantly correlated with the regular use of antipsychotics. In contrast, in the non-SSDs group, formula feeding was significantly correlated with the regular use of benzodiazepines, the number of benzodiazepines used, the diazepam equivalent, and the number of psychotropic drugs used. These findings suggest that the interventions required to achieve exclusive breastfeeding might differ between individuals with SSDs and those with non-SSDs.

We found that individuals with SSDs had lower exclusive breastfeeding rates than individuals with other psychiatric disorders did. Previous studies have shown that individuals with SSDs are at a greater risk of having low birth weight infants and neonatal complications than individuals with other psychiatric disorders are [44, 45], although no significant differences in the rate of low birth weight or neonatal complications were observed among our disorder groups. In general, low birth weight infants or neonatal complications are associated with a decrease in exclusive breastfeeding rates [46, 47]. Additionally, SSDs cause impairments in cognitive functions, such as processing speed, attention, working memory, and social cognition [48], and these cognitive impairments are more severe in individuals with SSDs than in those with other psychiatric disorders [49, 50]. Both neonatal conditions and individual cognitive functions might have contributed to the differences in the exclusive breastfeeding rates between the SSDs group and the non-SSDs group.

In the SSD group, the use of antipsychotics at the time of childbirth was significantly associated with formula feeding. SSDs are chronic disorders, and continued treatment with antipsychotics throughout the lifespan is recommended [51, 52]. Several guidelines by the WHO and the National Institute for Health and Care Excellence (NICE) encourage women with mental health problems to exclusively breastfeed, unless those with SSDs are taking clozapine [6, 32]. In contrast, the NICE guidelines suggest that when assessing the risks and benefits of antipsychotics for women who are exclusively breastfeeding, the following factors should be considered; the limited data on the safety of antipsychotics, the levels of antipsychotics in breast milk, and the need for monitoring adverse effects in the babies [32]. The RID is an indicator of the amount of drug an infant ingests via breast milk, and a lower RID is considered safer for infants. Some first-generation antipsychotics have a maximum RID of more than 10%, such as haloperidol (12%) [43], and the maximum RID for second-generation antipsychotics is less than 10%, e.g., olanzapine: 0.28–2.24%, risperidone: 2.8–9.1%, quetiapine: 0.02–0.1%, and aripiprazole: 0.7–6.44% [43]. Second-generation antipsychotics have a lower RID, making them safer for exclusive breastfeeding, and are recommended for use in individuals with SSDs who wish to exclusively breastfeed, although careful consideration of exclusive breastfeeding, such as monitoring adverse effects in the baby, is necessary.

Compared with the previously reported range of 37% to 48% in the general population [8–11], the exclusive breastfeeding rate, which ranged from 4% to 21%, in our non-SSD group (individuals with BDs, DDs, and ASRDs) was low. In the non-SSD group, the use of benzodiazepines and psychotropic medication polypharmacy significantly affected formula feeding. This result was not surprising, because the regular use of benzodiazepines during exclusive breastfeeding is not recommended, especially for the newborns and preterm infants of individuals with any psychiatric disorder [32]. Furthermore, polypharmacy with psychotropic drugs has been reported to be associated with a greater risk of sedation in infants than monotherapy [53]. This finding suggests that for individuals in the non-SSDs group, reducing or discontinuing regular benzodiazepine use and polypharmacy during the perinatal period might lead to an increase in the rate of exclusive breastfeeding.

In the SSD group, infant feeding methods were associated with the use of antipsychotics, while in the non-SSD group, they were associated with the use of benzodiazepines and the number of psychotropic drugs used. These findings suggest that the factors associated with infant feeding methods differ between the SSD and non-SSD groups. However, the types of psychotropic medications required differ between these groups. Therefore, a lower number or dosage of the required psychotropic medications may be commonly associated with exclusive breastfeeding in both groups.

There are several limitations to consider in the interpretation of our findings. Our participants’ data were collected from medical records at a single university hospital. Compared with individuals in general obstetric clinics, and general hospitals, the individuals with psychiatric disorders in university hospitals and their newborns might have more serious psychiatric and/or physical complications in university hospitals. Although lower education levels [54], lower intentions to breastfeed [38], and lower receipt of breastfeeding support, such as attendance at prenatal breastfeeding classes or peer counseling [38, 54], might have been associated with lower rates of exclusive breastfeeding, this study did not collect information on these factors. Additionally, we extracted data on infant feeding methods at one month postpartum; however, the infant feeding methods before and after that time are unknown. Future studies should collect data on long-term infant feeding methods across multiple facilities to investigate the effects of clinical factors on infant feeding practices among individuals with psychiatric disorders. In this study, the dependent variable, infant feeding method, was treated as a continuous variable (exclusive breastfeeding < mixed feeding < formula feeding) and applied linear regression, as these categories lie on a continuum and this approach avoids quasi-complete separation with the limited sample size. Meanwhile, to ensure robustness, we additionally performed ordinal logistic regression (Supplementary Fig. 1). As expected, however, some factors could not be evaluated due to quasi-complete separation. For the factors that could be analyzed, ordinal logistic regression yielded results consistent with the linear regression model, suggesting that our findings are not dependent on the analytic framework.

## Conclusion

This study revealed significant differences in infant feeding practices among individuals with psychiatric disorders. Individuals with SSDs are more likely to choose formula feeding than individuals with non-SSDs are. Formula feeding was affected by antipsychotic drug use in individuals with SSDs, whereas formula feeding was affected by psychotropic polypharmacy, including the use of benzodiazepines in individuals with non-SSDs. Providing safety education on antipsychotics for individuals with SSDs, avoiding psychotropic polypharmacy, particularly minimizing the regular use of benzodiazepines in non-SSDs patients, and fostering collaboration between psychiatry and obstetrics, could support a reduction in the rate of formula feeding, which may contribute to achieving exclusive breastfeeding.

## Supplementary Information

Below is the link to the electronic supplementary material.

Supplementary Material 1.

## Data Availability

No datasets were generated or analysed during the current study.
