# Supplementary material for: Differences in infant feeding methods at one month postpartum among women with psychiatric disorders and factors influencing exclusive breastfeeding
Source: Child Adolesc Psychiatry Ment Health. 2025 Oct 27;19:116. doi: 10.1186/s13034-025-00973-7 (PMC12557946; doi:10.1186/s13034-025-00973-7)
Supplement: Supplementary file 1 — Supplementary Material 1. [file 13034_2025_973_MOESM1_ESM.docx]

**Supplementary Information**

**Supplementary Table 1.** Differences in infant feeding methods at one month postpartum among the four disorder groups.

| Comparison | **Adjusted Mean Difference** [**95% CI]** | ***p* value** |
| --- | --- | --- |
| SSDs vs. BDs | 0.38 [0.017, 0.74] | **0.04** |
| SSDs vs. DDs | 0.68 [0.34, 1.01] | **9.41 × 10⁻⁵** |
| SSDs vs. ASRDs | 0.56 [0.27, 0.85] | **2.41 × 10⁻⁴** |

The *post-hoc* comparisons were conducted using ANCOVA to examine group differences in infant feeding methods at one month postpartum, adjusting for age and year of childbirth. Infant feeding methods were assigned numerical values as follows: 1 = exclusive breastfeeding, 2 = mixed feeding, and 3 = formula feeding. Adjusted mean differences represent estimated group differences based on this scale. SSDs, Schizophrenia spectrum disorders; BDs, bipolar disorders; DDs, depressive disorders; ASRDs, anxiety disorders and stress-related disorders. *P*<0.05 is shown in bold.

**Supplementary Table 2.** Factors influencing infant feeding methods in individuals schizophrenia spectrum disorders (SSDs).

| **SSDs** **(*n*=33)** | **Standardized *beta*** | **Unstandardized beta [95%CI]** | ***p* value** |
| --- | --- | --- | --- |
| Regular use of antipsychotics | 0.52 | 0.82 [0.31, 1.34] | **2.93×10^-3^***** |
| Number of antipsychotics | 0.47 | 0.50 [0.14, 0.85] | **7.94×10^-3^**** |
| Number of psychotropic drugs | 0.40 | 0.14 [0.016, 0.27] | **0.028*** |
| Current Smoker | 0.35 | 0.43 [-0.018, 0.87] | 0.059 |
| First pregnancy | 0.33 | 0.36 [-0.044, 0.75] | 0.069 |
| Hospitalization in the NICU | 0.31 | 0.40 [-0.075, 0.87] | 0.096 |
| Maximum RID | 0.26 | 0.03 [-0.016, 0.065] | 0.22 |
| Have an occupation | 0.26 | 0.46 [-0.23, 1.15] | 0.18 |
| Number of mood stabilizers | 0.22 | 0.48 [-0.35, 1.32] | 0.25 |
| Regular use of mood stabilizers | 0.22 | 0.48 [-0.35, 1.32] | 0.25 |
| CPZeq | 0.20 | 0.001 [-0.001, 0.002] | 0.31 |
| Comorbid physical disease | 0.19 | 0.31 [-0.32, 0.94] | 0.33 |
| Regular use of antidepressants | 0.16 | 0.35 [-0.49, 1.19] | 0.40 |
| IMIeq | 0.16 | 0.004 [-0.006, 0.013] | 0.41 |
| Number of benzodiazepines | 0.16 | 0.12 [-0.18, 0.42] | 0.42 |
| DZPeq | 0.16 | 0.02 [-0.026, 0.061] | 0.41 |
| Comorbid psychiatric disorders | 0.15 | 0.28 [-0.47, 1.02] | 0.45 |
| Regular use of benzodiazepines | 0.12 | 0.15 [-0.38, 0.68] | 0.56 |
| Number of antidepressants | 0.12 | 0.21 [-0.33, 0.74] | 0.53 |
| Multiple gestation | 0.11 | 0.23 [-0.64, 1.10] | 0.59 |
| Sex of the newborn | 0.08 | 0.09 [-0.34, 0.52] | 0.67 |
| Delivery method | 0.05 | 0.03 [-0.23, 0.29] | 0.81 |
| Physical complications related to pregnancy | -0.11 | -0.12 [-0.53, 0.30] | 0.56 |
| Apgar score at 1 minute | -0.13 | -0.03 [-0.13, 0.06] | 0.50 |
| Birth weight of the newborn | -0.14 | -0.0001 [-0.001, 0.000] | 0.48 |
| Apgar score at 5 minutes | -0.19 | -0.04 [-0.13, 0.045] | 0.34 |
| Married | -0.28 | -0.44 [-1.05, 0.17] | 0.15 |

NICU, neonatal intensive care unit; RID, relative infant dose; CPZeq, chlorpromazine equivalent; IMIeq, imipramine equivalent; DZPeq, diazepam equivalent. *** *P* <2.94×10^-3^, ** *P* <0.01, * *P* <0.05. *P*<0.05 is shown in bold.

**Supplementary Table 3.** Factors influencing infant feeding methods in individuals with non-schizophrenia spectrum disorders (non-SSDs).

| **non-SSDs** **(*n*=141)** | **Standardized *beta*** | **Unstandardized beta [95%CI]** | ***p* value** |
| --- | --- | --- | --- |
| Regular use of benzodiazepines | 0.43 | 0.72 [0.43, 1.02] | **3.46×10^-6^***** |
| Number of psychotropic drugs | 0.41 | 0.23 [0.13, 0.33] | **8.42×10^-6^***** |
| Number of benzodiazepines | 0.38 | 0.39 [0.21, 0.57] | **3.71×10^-5^***** |
| DZPeq | 0.31 | 0.028 [0.012, 0.043] | **6.78×10^-4^***** |
| Number of antidepressants | 0.26 | 0.34 [0.10, 0.58] | **5.50×10^-3^**** |
| Maximum RID | 0.25 | 0.032 [0.008, 0.056] | **0.010**** |
| Physical complications related to pregnancy | 0.21 | 0.30 [0.036, 0.56] | **0.026*** |
| Regular use of antidepressants | 0.21 | 0.35 [0.034, 0.66] | **0.030*** |
| Hospitalization in the NICU | 0.20 | 0.28 [0.026, 0.54] | **0.031*** |
| IMIeq | 0.20 | 0.003 [0.000, 0.005] | **0.037*** |
| Comorbid psychiatric disorders | 0.14 | 0.29 [-0.088, 0.66] | 0.13 |
| First pregnancy | 0.14 | 0.20 [-0.083, 0.48] | 0.17 |
| Number of antipsychotics | 0.12 | 0.20 [-0.12, 0.52] | 0.23 |
| Regular use of antipsychotics | 0.12 | 0.24 [-0.16, 0.64] | 0.24 |
| CPZeq | 0.10 | 0.001 [-0.001, 0.002] | 0.32 |
| Regular use of mood stabilizers | 0.083 | 0.27 [-0.27, 0.70] | 0.38 |
| Number of mood stabilizers | 0.083 | 0.27 [-0.27, 0.70] | 0.38 |
| Delivery methods | 0.047 | 0.054 [-0.16, 0.27] | 0.62 |
| Have an occupation | 0.016 | 0.026 [-0.29, 0.34] | 0.87 |
| Married | -0.013 | -0.040 [-0.63, 0.55] | 0.89 |
| Current Smoker | -0.029 | -0.15 [-1.16, 0.86] | 0.76 |
| Comorbid physical disorders | -0.041 | -0.063 [-0.35, 0.22] | 0.67 |
| Sex of the newborn | -0.088 | -0.12 [-0.39, 0.14] | 0.35 |
| Apgar score at 5 minutes | -0.14 | -0.12 [-0.29, 0.045] | 0.15 |
| Birth weight of the newborn | -0.16 | -0.0003 [-0.001, 0.000] | 0.092 |
| Apgar score at 1 minute | -0.21 | -0.17 [-0.32, -0.020] | **0.027*** |

DZPeq, diazepam equivalent; RID, relative infant dose; NICU, neonatal intensive care unit; IMIeq, imipramine equivalent; CPZeq, chlorpromazine equivalent. *** *P* <2.94×10^-3^, ** *P* <0.01, * *P* <0.05. *P*<0.05 is shown in bold.

**Supplementary Figure 1.** Factors associated with infant feeding methods in individuals with and without schizophrenia spectrum disorders assessed by ordinal logistic regression analyses.


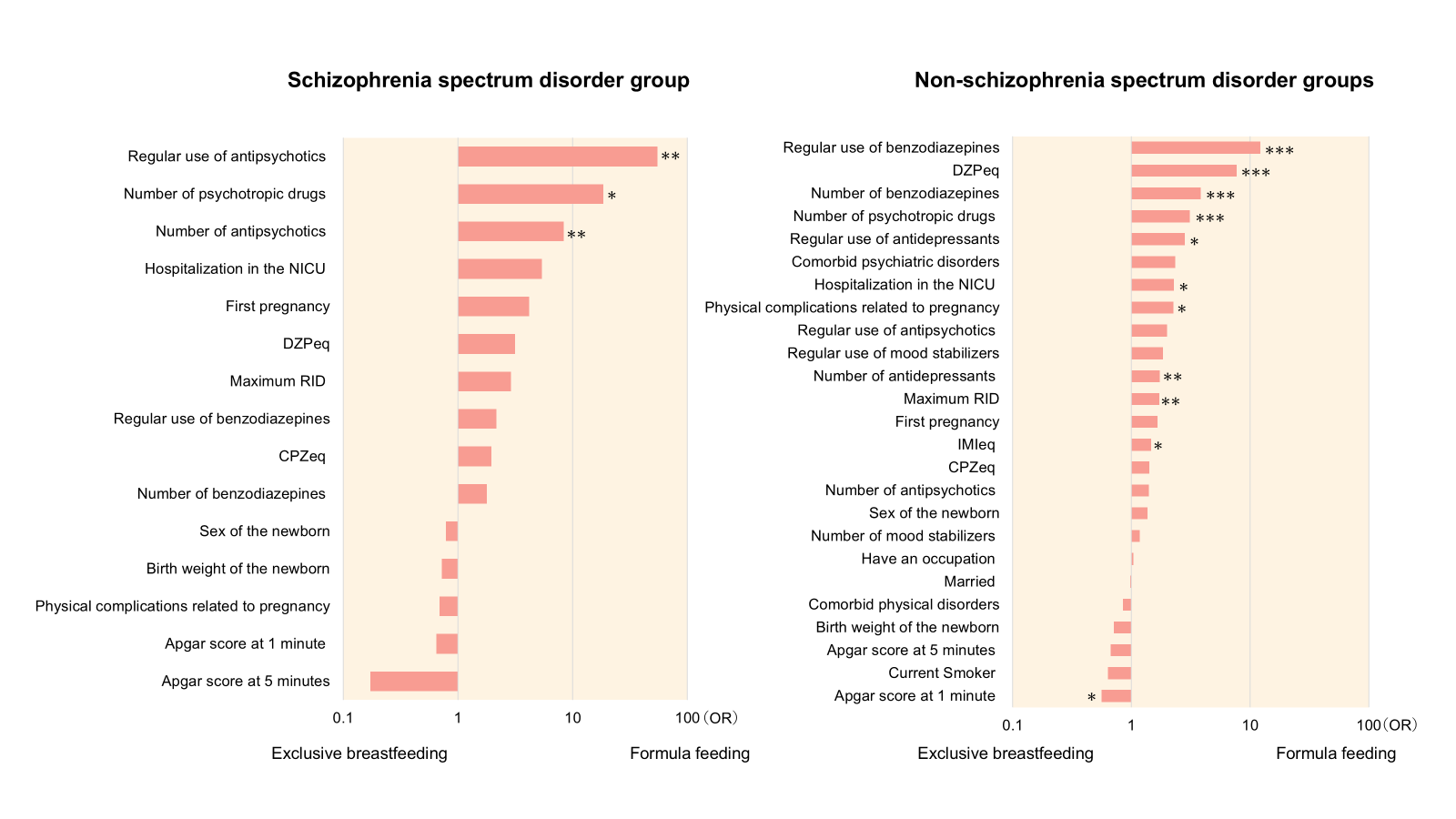


An ordinal logistic regression analysis was conducted with the infant feeding methods at one month postpartum (formula feeding: 3, mixed feeding: 2, exclusive breastfeeding: 1) as the dependent variable, individual and newborn variables as the independent variables, and age and year of childbirth as covariates. Odds ratios (ORs) obtained from ordinal logistic regression analyses are shown separately for individuals with and without schizophrenia spectrum disorders. Due to quasi-complete separation, odds ratios for some factors could not be estimated. *** *p*<2.94×10^-3^, ***p*<0.01, **p*<0.05. NICU, neonatal intensive care unit; DZPeq, diazepam equivalent; RID, relative infant dose; CPZeq, chlorpromazine equivalent; IMIeq, imipramine equivalent.
